# A smartphone-based test for the assessment of attention deficits in delirium: A case-control diagnostic test accuracy study in older hospitalised patients

**DOI:** 10.1371/journal.pone.0227471

**Published:** 2020-01-24

**Authors:** Zoë Tieges, David J. Stott, Robert Shaw, Elaine Tang, Lisa-Marie Rutter, Eva Nouzova, Nikki Duncan, Caoimhe Clarke, Christopher J. Weir, Valentina Assi, Hannah Ensor, Jennifer H. Barnett, Jonathan Evans, Samantha Green, Kirsty Hendry, Meigan Thomson, Jenny McKeever, Duncan G. Middleton, Stuart Parks, Tim Walsh, Alexander J. Weir, Elizabeth Wilson, Tara Quasim, Alasdair M. J. MacLullich

**Affiliations:** 1 Edinburgh Delirium Research Group, University of Edinburgh, Edinburgh, Scotland, United Kingdom; 2 Centre for Cognitive Ageing and Cognitive Epidemiology, University of Edinburgh, Scotland, Edinburgh, United Kingdom; 3 Institute of Cardiovascular and Medical Sciences, University of Glasgow, Glasgow, Scotland, United Kingdom; 4 Edinburgh Clinical Trials Unit, Usher Institute of Population Health Sciences and Informatics, University of Edinburgh, Edinburgh, United Kingdom; 5 Cambridge Cognition Ltd, Cambridge, United Kingdom; 6 Department of Psychiatry, University of Cambridge, Cambridge, United Kingdom; 7 Institute of Health and Wellbeing, University of Glasgow, Glasgow, Scotland, United Kingdom; 8 Medical Devices Unit, West Glasgow Ambulatory Care Hospital, Glasgow, Scotland, United Kingdom; 9 Critical Care Medicine and Anaesthesia, University of Edinburgh, Edinburgh, Scotland, United Kingdom; 10 Critical Care Medicine and Anaesthesia, Royal Infirmary of Edinburgh, Edinburgh, Scotland, United Kingdom; 11 Anaesthesia, Critical Care and Pain Medicine, Glasgow Royal Infirmary, Glasgow, Scotland, United Kingdom; University of California, San Francisco, UNITED STATES

## Abstract

**Background:**

Delirium is a common and serious acute neuropsychiatric syndrome which is often missed in routine clinical care. Inattention is the core cognitive feature. Diagnostic test accuracy (including cut-points) of a smartphone Delirium App (DelApp) for assessing attention deficits was assessed in older hospital inpatients.

**Methods:**

This was a case-control study of hospitalised patients aged ≥65 years with delirium (with or without pre-existing cognitive impairment), who were compared to patients with dementia without delirium, and patients without cognitive impairment. Reference standard delirium assessment, which included a neuropsychological test battery, was based on Diagnostic and Statistical Manual of Mental Disorders-5 criteria. A separate blinded assessor administered the DelApp arousal assessment (score 0–4) and attention task (0–6) yielding an overall score of 0 to 10 (lower scores indicate poorer performance). Analyses included receiver operating characteristic curves and sensitivity and specificity. Optimal cut-points for delirium detection were determined using Youden’s index.

**Results:**

A total of 187 patients were recruited, mean age 83.8 (range 67–98) years, 152 (81%) women; n = 61 with delirium; n = 61 with dementia without delirium; and n = 65 without cognitive impairment. Patients with delirium performed poorly on the DelApp (median score = 4/10; inter-quartile range 3.0, 5.5) compared to patients with dementia (9.0; 5.5, 10.0) and those without cognitive impairment (10.0; 10.0, 10.0). Area under the curve for detecting delirium was 0.89 (95% Confidence Interval 0.84, 0.94). At an optimal cut-point of ≤8, sensitivity was 91.7% (84.7%, 98.7%) and specificity 74.2% (66.5%, 81.9%) for discriminating delirium from the other groups. Specificity was 68.3% (56.6%, 80.1%) for discriminating delirium from dementia (cut-point ≤6).

**Conclusion:**

Patients with delirium (with or without pre-existing cognitive impairment) perform poorly on the DelApp compared to patients with dementia and those without cognitive impairment. A cut-point of ≤8/10 is suggested as having optimal sensitivity and specificity. The DelApp is a promising tool for assessment of attention deficits associated with delirium in older hospitalised adults, many of whom have prior cognitive impairment, and should be further validated in representative patient cohorts.

## Introduction

Delirium is a common and serious neuropsychiatric syndrome with core features of impaired attention, altered arousal and global cognitive dysfunction. It is mostly triggered by acute illness, trauma or medications. Delirium affects at least 1 in 6 older hospital patients [1, 2] and is associated with multiple adverse outcomes including higher mortality, new institutionalisation and increased risk of dementia [1, 3, 4]. Delirium is often highly distressing for patients and their carers [5, 6].

Despite its frequency and seriousness, delirium is often not detected in hospital due to its variable presentation, fluctuating nature of symptoms and an under-appreciation of its significance by healthcare providers [7]. This presents a barrier to improved care, because accurate diagnosis is an important step towards prompt management of underlying causes, relief of distress, and good communication with patients and carers [8]. Improving the detection of delirium is a priority for healthcare systems in the UK and internationally [9, 10].

A core diagnostic feature of delirium is impaired attention [11]. Assessment of attention is not only essential for diagnosing delirium, but is also important for differentiating delirium from dementia [12]. These syndromes have considerable symptom overlap and can exist simultaneously in the same patient [13, 14], but attention deficits are usually more marked in delirium [15, 16].

Attention deficits are usually assessed using either patient interview or formal cognitive testing, or a combination of both. Most studies show that inter-rater reliability for subjective assessments of inattention is low-to-moderate [17–19].

Several neuropsychological tests are in use to objectively assess inattention in delirium (such as months of the year backward or digit span) [15]. The available evidence suggests that most attention tests are sensitive to delirium, and that attention deficits are often greater in delirium compared to dementia (at least in the milder stages) but with varying degrees of overlap in scores [12, 20]. Indeed, some studies have shown impairments on bedside tests of attention in dementia, including spatial span [13] and months of the year backwards [21]. Formal validation studies of these tests for detecting delirium and discriminating delirium from dementia and other disorders common in older people are sparse. The lack of well-validated objective assessment tools for inattention in delirium leads to uncertainty over diagnosis, which may contribute to the low rate of delirium detection.

To address this gap, we developed a new objective neuropsychological test specifically designed for bedside assessment of attentional function in delirium, the Delirium App (DelApp). The DelApp provides an objective, standardised bedside assessment of the presence and degree of attention deficits characteristic of delirium (i.e. deficits in sustained and focused attention, and basic orienting of attention [12, 16]). The DelApp yields a score in patients too unwell or drowsy to undergo interview or formal cognitive testing and therefore no patients are classed as ‘unable to assess’ (a known problem of many delirium assessment tools [22]).

Proof-of-principle studies (single-rater) of DelApp and similar tasks administered first via an electronic test box (Edinburgh Delirium Test Box) and then a smartphone-based version suggest that this test performs well as a method for objectively assessing attention in delirium and for discriminating between delirium (with or without prior cognitive impairment) and dementia [12, 23, 24]. They also suggest that the DelApp is practical and acceptable to patients.

Here we performed a case-control study to evaluate the potential diagnostic performance (sensitivity, specificity) of the DelApp as an instrument for detecting attention deficits in delirium in older hospitalised patients, and to determine optimal cut-points.

## Methods

### Study design

This was a case-control study involving patients recruited from general and acute medical hospital wards in the Glasgow Royal Infirmary, Royal Infirmary of Edinburgh, and rehabilitation wards in Liberton Hospital in Edinburgh between 28^th^ October 2015 and 5^th^ April 2016. Cases and controls were frequency-matched by age within 10-year age bands and sex. Three groups of patients were recruited: patients with delirium (with or without dementia), patients with a diagnosis of dementia (without delirium), and patients without cognitive impairment. Research ethics approval was obtained from the Scotland A Research Ethics Committee (reference 15/SS/0104). The study was registered on a clinical trial database (http://clinicaltrials.gov, reference number NCT02590796) and approved by the Medicines and Healthcare products Regulatory Agency (MHRA, reference CI/2015/0031).

### Participants

Patients aged 65 years or older who were admitted to a general or acute medical hospital ward were eligible. Where the participants lacked capacity to give informed consent, an appropriate personal or nominated consultee, guardian, welfare attorney or nearest relative was contacted to provide informed consent. Exclusion criteria were: unable to understand spoken English, severe visual or hearing impairment and photosensitive epilepsy. Potentially eligible patients were identified by discussing the selection criteria with a clinician responsible for the patient’s care.

### Measurements and Procedures

Assessment of cognitive function and delirium were conducted by trained assessors who were all psychology graduates (EN, LMR, ND, CC and ZT). They received comprehensive training from senior geriatricians (AMJM and DJS) prior to the start of the study, which included training videos, introductory visits to the hospital wards, observing clinicians performing the diagnostic work-up for delirium, and role-play. The diagnostic performance of the DelApp (index test) was evaluated against a reference standard diagnosis of delirium based on Diagnostic and Statistical Manual of Mental Disorders-5 (DSM-5) criteria [25]. Reference standard and DelApp assessments were administered to the same patients by pairs of assessors. The assessor who administered the DelApp test was blinded to the reference standard assessment, the patients’ diagnosis and other clinical information to ensure unbiased scoring of the DelApp. The target interval between index and reference standard assessments was 15–60 min with a maximum possible interval of 3 hours.

#### DelApp

The DelApp has been described in detail in Rutter et al. [26]. It is a smartphone-based test comprising a brief arousal assessment followed by a sustained attention task. The arousal assessment was developed to provide some grading of DelApp test scores in patients unable to perform the attention task. This incorporates assessment of basic alertness and orienting which is also sometimes referred to as lower-level attention processes [27]. Specifically, the arousal assessment involves judging whether the patient is awake and responsive and is able to open their eyes for more than 10 s (2 points) or less than 10 s (1 point); asking the patient to say their name or obey a one-stage command (e.g., lifting one arm) (1 point); and asking the patient to follow an object with their eyes for 5 s (1 point). This yields a maximum possible score of 4 which indicates that the patient is awake and able to follow basic commands. Participants who achieve a score of ≥3 on the arousal assessment proceed with the attention task. In case of an arousal score below 3, the assessment ends and the participant obtains a total DelApp score based on the arousal assessment alone.

The attention task requires counting a series of large white five-pointed stars appearing on the smartphone screen against a black background. To increase attentional load, as the task progresses, distracting triangular shapes appear around the stimuli and the inter-stimulus interval between stars lengthens. The counting task consists of 7 trials, the first being a practice trial which is not scored. Participants are asked to count and then verbally report at the conclusion of the sequence (as indicated by the examiner) the number of times they saw the stars presented on the smartphone screen. Trials are scored as correct or incorrect, with missing answers considered incorrect responses, and the test ends after two consecutive incorrect responses. The total DelApp score is the sum of the arousal score (score 0–4) and attention score (score 0–6), yielding a total between 0 and 10 (10 = best possible performance). The DelApp takes five minutes or less to complete.

#### Reference standard assessment

The reference standard assessment has been described elsewhere [26]. In summary, the following assessment tools were used: the short Orientation-Memory-Concentration test (OMCT), a six-item cognitive test focused on orientation and working memory (score range 0–28, score of 20 or below indicates cognitive impairment) [28]; attention tests including digit span forwards and backwards, days of the week and months of the year backwards [29]; and the Delirium Rating Scale-Revised-98 (DRS-R98 [30]) to aid delirium assessment, supplemented by the Observational Scale of Level of Arousal (OSLA [24]) and the Richmond Agitation-Sedation Scale (RASS [31, 32]) for measuring level of arousal. From the 58^th^ participant onwards, two further assessment tools were included in the reference standard assessment battery: the Vigilance A task [33] and a pain rating scale (available online at https://uvahealth.com/sites/default/files/2018-07/PE15019C-UVAPainRatingScale.pdf (Accessed on 18 December 2018)), to assess patients’ pain intensity (score range 0–10, 10 = worst possible pain). The reference standard assessment lasted approximately 20 minutes.

### Grouping of participants

Delirium was ascertained according to DSM-5 diagnostic criteria, informed by results from the reference standard assessment alongside observational and medical information to determine group allocation (described in [26]). Where initial grouping based on information provided by the patient’s clinical team and medical notes was not consistent with the outcomes from the reference standard assessment, the most appropriate grouping for these cases was decided blind to DelApp results by a consultant geriatrician (AMJM or DJS) based on all the other available information (i.e. cognitive test scores, medical notes and information from clinical team). Dementia diagnosis was ascertained through medical notes and/or discussion with the clinical team. Patients were categorized as having dementia (without delirium) in case of a documented, formal prior diagnosis of dementia. Patients for whom an OMCT score >20 was obtained and who did not have an acute change from baseline or a diagnosis of dementia were grouped as having no cognitive impairment. Patients who could not be allocated to any group, because they did not present a symptom profile that was characteristic of any one of the pre-specified groups, were termed ‘indeterminate’ and excluded from the analysis (in advance of knowledge of DelApp scores).

### Sample size calculation

The sample size required was determined using a normal approximation to the binomial distribution to estimate a 95% confidence interval (CI) for measures of diagnostic accuracy for delirium. With delirium and comparator group sizes of 50, sensitivity and specificity for the delirium versus no delirium comparison and the delirium versus dementia comparison can be estimated precisely when the diagnostic performance is good (sensitivity or specificity equal to 90%; confidence interval width ±8.3%), and moderately precisely in other scenarios (sensitivity or specificity between 50% and 70%; confidence interval widths between ±12.7 and ±13.9).

The recruitment target was set at N = 60 per clinical group (instead of N = 50), as it was anticipated that some patients would be re-grouped as indeterminate following the reference standard assessment, and thereby excluded from the final analyses.

### Statistical analysis

Measures of diagnostic accuracy of the DelApp were determined by receiver operating characteristic (ROC) curve analysis. Sensitivity and specificity for delirium detection were calculated for the whole sample (delirium, dementia and cognitively unimpaired) and for delirium versus dementia groups. Optimal cut-off values were determined using Youden’s index. Positive and negative predictive values were also calculated, with the caveat that these depend on the prevalence of delirium which is artificially controlled by the case-control design.

Spearman correlations were used to evaluate construct validity via the association between DelApp scores with delirium symptom severity (DRS-R98) scores, and scores on other tests of attention: digit span, months of the year backward and days of the week backwards.

Participants with missing data on the primary outcome measures or those grouped as indeterminate were excluded from statistical analysis. All statistical tests were two-sided and performed using a 0.05 significance level; 95% CIs are presented.

The study sample included a wide range of severity of cognitive impairment. Hence, a post-hoc ROC analysis was conducted stratifying the sample by cognitive impairment using OMCT score categories (severe cognitive impairment: score 0–8; mild-to-moderate impairment: score 9–20; cognitively normal: score >20) to explore diagnostic performance of DelApp in subgroups of severity of cognitive impairment.

A further post-hoc analysis was conducted to explore diagnostic performance of the DelApp for detecting delirium in those without a dementia diagnosis.

## Results

### Participants

**Fig 1:**
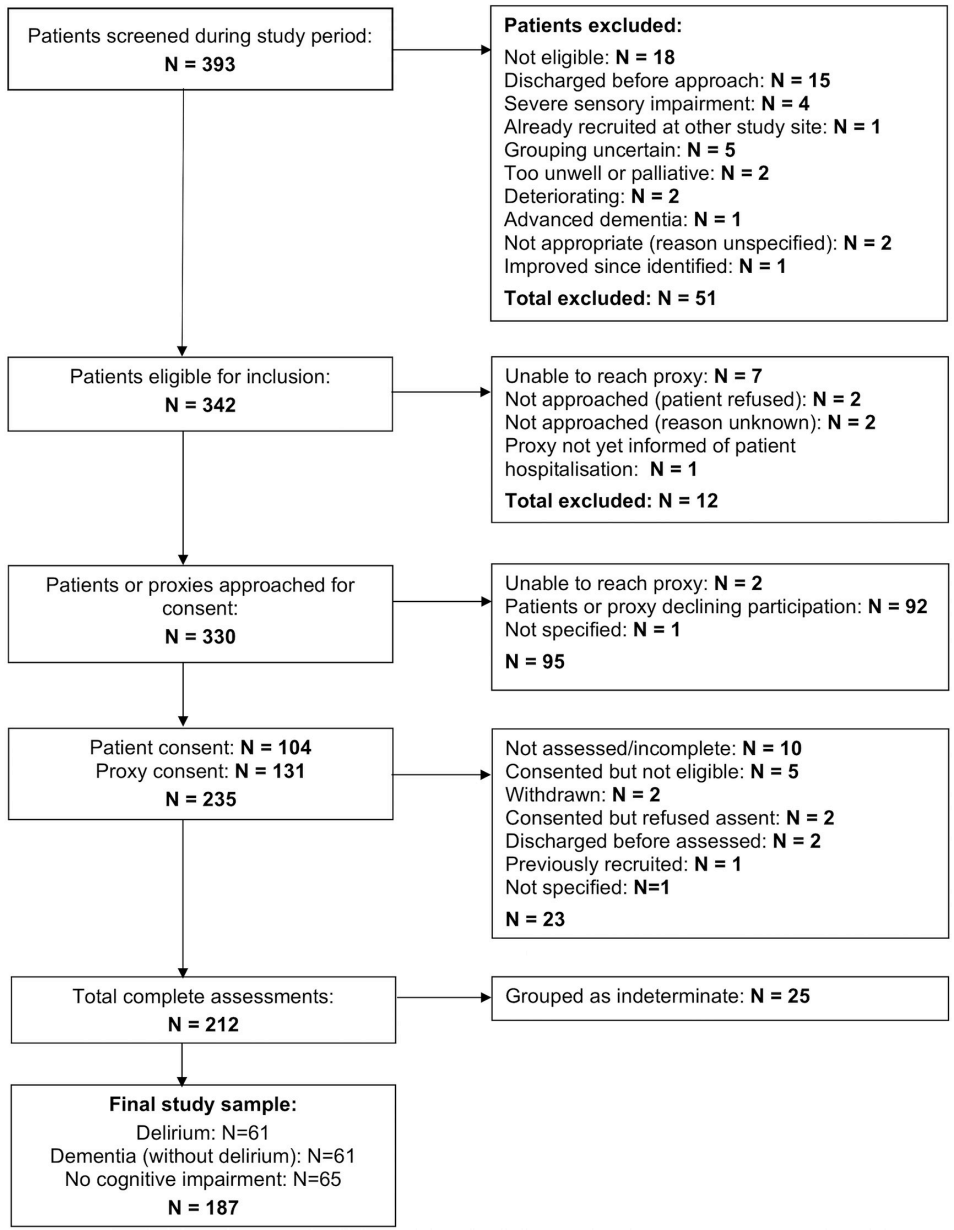
Patient recruitment flowchart.

A total of 342 patients were eligible for study inclusion. Of these, informed consent was obtained from 235 patients or their proxies, and 212 patients completed the study assessments. Twenty-five patients were grouped as indeterminate (because they could not be allocated to any of the clinical groups) and therefore excluded from data analysis, yielding a final study sample of 187 patients (n = 61 delirium with or without pre-existing cognitive impairment including dementia; n = 61 dementia without delirium; and n = 65 no cognitive impairment; Fig 1). Thirty-one (50.8%) patients in the delirium group also had a formal diagnosis of dementia. Mean age of the final study sample was 83.8 (range 67–98) years and 152 (81.3%) were women (Table 1).

**Table 1:** Patient characteristics.

	Total	Cases: Delirium (with or without pre-existing cognitive impairment)	Controls: Dementia (no delirium)	Controls: No cognitive impairment
**Number**	187	61	61	65
**Sex (female) (number (%))**	152/187 (81.3%)	49/61 (80.3%)	45/61 (73.8%)	58/65 (89.2%)
**Age (years) (mean ±S.D.)**	83.8 (±6.6) (n = 187)	85.8 (±6.1) (n = 61)	85.3 (±5.6) (n = 61)	80.4 (±6.7) (n = 65)
**Current smoker** **(number (%))**	8/141 (5.7%)	6/42 (14.3%)	2/49 (4.1%)	0/50 (0.0%)
**Medications (number) (mean ±S.D.)**	7.9 (±4.3) (n = 173)	7.8 (±3.8) (n = 55)	7.3 (±4.2) (n = 56)	8.4 (±4.7) (n = 62)
**Charlson Co-morbidity score (mean ±S.D.)**	3.3 (±2.3) (n = 183)	3.8 (±2.5) (n = 60)	3.4 (±2.3) (n = 61)	2.8 (±2.1) (n = 62)
**Length of stay (days) (median, IQR)**	16 (11–30.5) (n = 172)	19 (12–35) (n = 51)	18 (11–36) (n = 59)	12.5 (8–23) (n = 62)
**Mortality at 12 weeks (number (%))**	24/184 (13.0%)	12/60 (20.0%)	8/61 (13.1%)	4/63 (6.3%)
**Short OMCT (score) (median, IQR)** ***Normal (N*, *%)*** ***Minimal cognitive impairment (N*, *%)*** ***Severe cognitive impairment (N*, *%)***	11 (3–25) (n = 182)	3 (0–7) (n = 58) 3 (5.2%) 9 (15.5%) 46 (79.3%)	6 (3–12) (n = 58) 6 (10.3%) 19 (32.8%) 33 (56.9%)	26 (23.5–28) (n = 65) 64 (98.5%) 1 (1.5%) 0 (0%)
**Months backward (7+ months correct) (number (%))**	67/167 (40.1%)	2/45 (4.4%)	13/58 (22.4%)	52/64 (81.3%)
**AMT-10 (score) (median, IQR)**	5 (2–9) (n = 178)	1 (0–4) (n = 56)	3 (2.5–5.5) (n = 57)	9 (8–10) (n = 65)
**Brief Attention Test (score) (median, IQR)**	5 (3–6) (n = 179)	2 (0–4) (n = 57)	4 (3–5) (n = 59)	7 (6–7) (n = 63)
**DRS-R98 total (score) (median, IQR)**	8 (1–17) (n = 185)	19 (15.5–23) (n = 60)	8 (6–11.5) (n = 60)	1 (0–1) (n = 65)
**DRS-R98 severity (score) (median, IQR)**	7 (1–13) (n = 186)	15 (10–19) (n = 61)	7.5 (6–11) (n = 60)	1 (0–1) (n = 65)
**OSLA (score) (median, IQR)**	0 (0–2) (n = 187)	3 (1–6) (n = 61)	0 (0–1) (n = 61)	0 (0–0) (n = 65)

Notes: Short OMCT = Short Orientation-Memory-Concentration Test (score range 0–28). Short OMCT categories: severe cognitive impairment (score 0–8), minimal impairment (score 9–20), normal (score >20). AMT10 = Abbreviated Mental Test-10 (score 0–10, score ≤7 indicates cognitive impairment). The Brief Attention Test comprises digit span (3 forward trials, 2 backward trials), months of the year backward and days of the week backward (total score range 0–7, score <5 indicates attention impairment). DRS-R98 = Delirium Rating Scale-Revised 98 (total score range 0–46 and severity sub-score range 0–39, higher scores indicate increased likelihood and severity of delirium). OSLA = Observational Scale of Level of Arousal (score range 0–15, higher scores indicate more abnormal level of arousal, incorporating both reduced and increased arousal). One participant was grouped as cognitively unimpaired despite an OMCT score indicating minimal cognitive impairment. Grouping was based on discussions with a senior clinician (AMJM) taking account of the overall neuropsychological score profile and researcher observations (protocol deviation).

### Diagnostic characteristics of DelApp

Patients with delirium performed more poorly on the DelApp (median score 4.0/10.0; inter-quartile range (IQR) 3.0, 5.5) compared to patients with dementia without delirium (median 9.0; IQR 5.5, 10.0) and those without cognitive impairment (median 10.0; IQR 10.0, 10.0).

**Fig 2:**
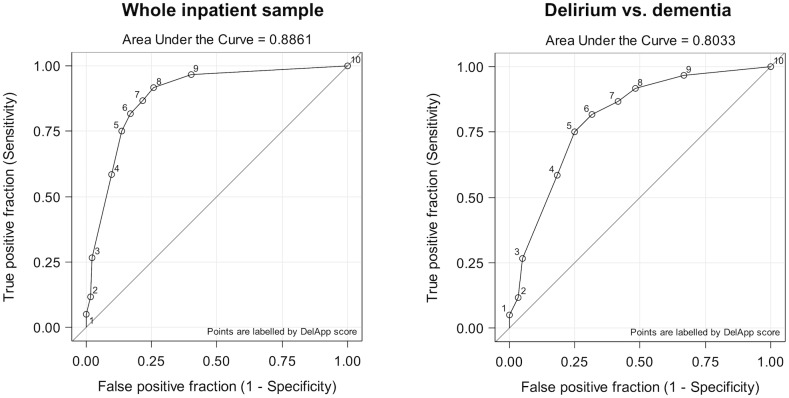
Receiver Operating Characteristic curves for DelApp for detecting DSM-5 delirium in the whole sample (i.e. delirium, dementia and cognitively normal groups; left panel) and for discriminating between delirium (with or without dementia) and dementia groups (right panel).

The area under the ROC curve for detecting delirium was 0.89 (95% CI [0.84, 0.94]). At an optimal cut-point of ≤8, sensitivity was 91.7% (95% CI [84.7%, 98.7%]) and specificity 74.2% (95% CI [66.5%, 81.9%]) for discriminating delirium in the whole sample (Fig 2, left panel). For the delirium versus dementia group comparison, the area under the ROC curve was 0.80 (95% CI [0.72, 0.88]), with a specificity for discriminating delirium from dementia of 68.3% (95% CI [56.6%, 80.1%]) at an optimal cut-point ≤6 (Fig 2, right panel). Sensitivity, specificity and Youden’s index for the different DelApp score cut-points are shown in Table 2 (S1 Table: positive and negative predictive values).

**Table 2:** Sensitivity, specificity and Youden’s index for different DelApp score cut-points.

	Delirium vs. inpatient sample			Delirium vs. dementia		
DelApp score	Sensitivity	Specificity	Youden’s Index	Sensitivity	Specificity	Youden’s Index
**0**	1.7%	100.0%	0.02	1.7%	100.0%	0.02
**1**	5.0%	100.0%	0.05	5.0%	100.0%	0.05
**2**	11.7%	98.4%	0.10	11.7%	96.7%	0.08
**3**	26.7%	97.6%	0.24	26.7%	95.0%	0.22
**4**	58.3%	90.3%	0.49	58.3%	81.7%	0.40
**5**	75.0%	86.3%	0.61	75.0%	75.0%	0.50
**6**	81.7%	83.1%	0.65	81.7%	68.3%	0.50
**7**	86.7%	78.2%	0.65	86.7%	58.3%	0.45
**8**	91.7%	74.2%	0.66	91.7%	51.7%	0.43
**9**	96.7%	59.7%	0.56	96.7%	33.3%	0.30
**10**	100.0%	0.0%	0.00	100.0%	0.0%	0.00

The sensitivity and specificity of the DelApp for detecting delirium in patients without a formal diagnosis of dementia (at a cut-point of ≤8) was 89.7% (95% CI [72.7%, 97.8%]) and 95.4% (95% CI [87.1%, 99.0%]), respectively (S2 Table and S1 Fig).

### Post-hoc analyses: Diagnostic characteristics of DelApp stratified by severity of cognitive impairment

Using the optimal cut-point of ≤6 as determined from the original ROC analysis, post-hoc analyses showed that specificity to distinguish delirium from dementia in a subgroup of patients with severe cognitive impairment (OMCT score 0–8; N total (delirium and dementia groups combined) = 77, N delirium = 45, N dementia = 32) was moderate at 59.4%. In patients with minimal or no cognitive impairment (short OMCT score 9–28; N total = 37, N delirium = 12, N dementia = 25) specificity for delirium versus dementia was 84.0%.

### Associations between DelApp with measures of delirium symptom severity and attentional function

Moderate-to-strong associations were found between DelApp with measures of general cognitive function, attention and arousal (Table 3). Lower DelApp scores (reflecting poorer test performance) were correlated with worse cognition as reflected in scores on the OMCT and AMT-10, worse attentional function and more abnormal arousal. DelApp scores were also associated with greater delirium symptom severity as assessed by the DRS-R98.

**Table 3:** Spearman correlations between DelApp with conventional measures of attention, arousal and delirium presence and symptom severity.

	N	Coefficient (r_s_)	95% confidence interval	p-value
Short OMCT	115	0.51	0.35, 0.63	<0.0001
AMT-10	111	0.46	0.30, 0.60	<0.0001
Brief Attention Test	115	0.60	0.46, 0.70	<0.0001
Months backward	102	0.36	0.18, 0.52	<0.0001
OSLA	120	-0.58	-0.68, -0.44	<0.0001
DRS-R98 severity	119	-0.50	-0.62, -0.35	<0.0001
DRS-R98 total	119	-0.60	-0.71, -0.47	<0.0001

Notes: Correlational analyses were conducted for groups with delirium and/or dementia. Short OMCT = Short Orientation Memory and Concentration Test; AMT-10 = Abbreviated Mental Task 10; DRS-R98 = Delirium Rating Scale-Revised 98; OSLA = Observational Scale of Level of Arousal. r_s_ = Spearman correlation coefficient.

## Discussion

This study found that the DelApp smartphone-based test had a sensitivity of 91.7% and specificity of 74.2% to delirium in the sample as a whole. The sample included a high proportion of people with prior cognitive impairment including dementia with over half of patients with delirium having a formal diagnosis of dementia. The area under the ROC curve was high at 0.89. Patients with delirium performed poorly on the DelApp compared to delirium-free individuals with dementia as well as those with no cognitive impairment. A cut-point of ≤8/10 was found (using Youden’s index) to have optimal sensitivity and specificity for identifying delirium. Moderate-to-strong associations between DelApp scores and measures of attention and delirium severity were found, providing evidence of the construct validity of the DelApp assessment. Overall, these findings suggest that DelApp is a promising tool for the objective assessment of inattention associated with delirium in older hospitalised patients.

The high sensitivity (>90%) of DelApp to delirium confirms findings from the preliminary single-rater case-control study [23], but the specificity of 68.3% for discriminating between delirium and dementia groups falls below the previously found value (87%). This likely reflects the large number of patients with moderate-to-severe cognitive impairment in the sample (43.1% according to OMCT scores) and a higher overall level of cognitive impairment in the dementia group in the present study (median OMCT score = 6 indicating severe cognitive impairment) compared to the preliminary study (median score = 12 indicating minimal cognitive impairment). Post-hoc analyses exploring the effect of severity of cognitive impairment suggest that severe cognitive impairment is associated with reduced specificity of the DelApp to distinguish delirium from dementia. Of note, studies in delirium patients have mostly not addressed the issue of dementia severity in comparison groups. Indeed, a systematic review of studies of delirium tools that included individuals with dementia found that none of the studies reported any effects of dementia severity or subtype [34]. Because severity of dementia affects performance on cognitive tests in general, including attention tests [14, 35], the lack of dementia severity reporting in such studies limits our ability to draw conclusions about the clinical utility of such tests in discriminating between delirium and dementia.

Another issue is that patients with severe dementia are often deemed ‘untestable’ using conventional cognitive tests such as the Mini-Mental State Examination [36]. These measurements are designed to assess patients in the milder stages of dementia and frequently show floor effects in patients with severe dementia, often because patients are too impaired to interact with the interviewer sufficiently to allow cognitive testing [37]. Therefore, some patients with severe dementia cannot be distinguished reliably from delirium using cognitive testing alone. Similar findings have been reported by Voyer and colleagues [38]. These authors found that, out of ten brief attention tests, the months of the year backwards test showed the best balance of sensitivity (83%) and specificity (63%) to delirium in older adults, however the specificity dropped to 19% in a subgroup of patients with premorbid cognitive impairment.

The overlap in cognitive test deficits in dementia and delirium means that such tests alone may be less useful in making a new diagnosis of delirium, especially when the dementia is severe [15, 39]. Thus, features such as onset and fluctuation and altered arousal provide the key data informing the diagnostic process [40]. Formal cognitive testing therefore has a particular role in detecting delirium in relation to patients with no cognitive impairment and mild-moderate dementia, and in detecting delirium superimposed upon mild-moderate dementia. The combined sensitivity and specificity of DelApp (which incorporates arousal and attention testing) in the subgroup with severe cognitive impairment suggests that DelApp may have value in informing the diagnostic process even in this challenging population.

This study has several strengths. The reference standard and DelApp assessments were performed by pairs of blinded raters, thereby minimising diagnostic review bias. Explicit and rigorous operationalised diagnostic criteria (i.e. reference standard) were used, incorporating several cognitive tests and observational scales and also a detailed delirium assessment instrument, the DRS-R98 [30]. Researchers were formally trained in the use of the reference standard and DelApp assessments, and throughout study recruitment remained under close supervision of two geriatricians and a research fellow, all with expertise in delirium assessment. The present study had clear, transparent selection criteria for group allocation and few exclusion criteria in order to permit inclusion of patients with a wide spectrum of cognitive ability and reduced level of arousal.

Several study limitations must also be acknowledged. Around half of the patients in the delirium group had a diagnosis of dementia, which precluded a specific investigation of the diagnostic accuracy of the DelApp for detecting delirium in the absence of dementia. Our reason for grouping delirious patients with and without dementia together was that the majority of older patients with delirium have underlying cognitive impairment or (often undiagnosed) dementia. Another reason for the design is pragmatic: it is challenging to recruit older patients with delirium who are known not to have any prior cognitive impairment, because one cannot exclude with certainty undiagnosed chronic cognitive impairment in the presence of delirium. Further, we did not have information regarding the severity of dementia in those patients with co-morbid delirium-dementia.

The case-control design is an important first step in assessing the potential diagnostic test accuracy, but cannot give a definitive evaluation of test validity as this method will exaggerate the performance of the tests compared to unselected cohorts. Another limitation concerns the use of OMCT scores to stratify patients by level of cognitive impairment for subgroup analysis. Whilst this test provides a measure of general cognition which is brief and suitable for use at the bedside, it may to some extent have been influenced by factors relating to acute illness, drugs and/or the hospitalisation itself. Future studies should consider including retrospective informant questionnaires such as the Informant Questionnaire of Cognitive Decline in the Elderly (IQCODE [41]) and/or an informant-based dementia severity rating instrument, in order to obtain more accurate information about the presence and severity of pre-existing cognitive impairment prior to admission.

Future studies are now needed to confirm diagnostic test accuracy using the suggested cut-points from the present study in a representative population of unselected older hospital inpatients, and to assess inter-rater reliability. Further, the DelApp provides a graded measure of attention reflecting the continuum of arousal and attentional impairment, which may be useful for detecting delirium at prodromal stages and tracking recovery from delirium following diagnosis. These questions could be addressed in future studies using within-patient longitudinal assessments of DelApp and delirium features. A more detailed characterisation of type and severity of pre-existing cognitive impairment and dementia would permit more thorough investigation of the diagnostic performance of DelApp in dementia subgroups. The present work highlights the need for further evaluation of the performance of the DelApp in dementia-free populations, for example in patients undergoing elective cardiothoracic surgery in whom pre-surgery cognitive assessment could be carried out. Future studies should also evaluate the practical use of DelApp (e.g. routine assessment by nurses), barriers to implementation, and, ultimately, benefit to patients via improved recognition of delirium and monitoring response to treatment. Finally, studies could seek to compare performance of DelApp with other delirium assessment tests such as the Delirium Observation Screening Scale [42] or the 3D-CAM [43], against an independent reference standard based on DSM or ICD criteria.

## Supporting information

S1 Table Positive and negative predictive values for DelApp. (DOCX)

S2 Table Sensitivity, specificity and Youden’s index for different DelApp score cut-points, for patients with delirium without a formal diagnosis of dementia vs patients without cognitive impairment. (DOCX)

S1 Fig Receiver Operating Characteristic curve for DelApp for detecting DSM-5 delirium in patients without a formal diagnosis of dementia. (DOCX)
