# Supplementary material for: A smartphone-based test for the assessment of attention deficits in delirium: A case-control diagnostic test accuracy study in older hospitalised patients
Source: PLoS One. 2020 Jan 24;15(1):e0227471. doi: 10.1371/journal.pone.0227471 (PMC6980392; doi:10.1371/journal.pone.0227471)
Supplement: S1 Table — (DOCX) [file pone.0227471.s001.docx]

S1 Table. Positive and negative predictive values for DelApp scores.

|  | Delirium vs. inpatient sample | Delirium vs. dementia |
| --- | --- | --- |
| Positive predictive value (95% CI) | 63.2% (53.1%, 73.4%) | 72.1% (61.4%, 82.7%) |
| Negative predictive value (95% CI) | 94.9% (90.4%, 99.3%) | 78.9% (67.8%, 90.0%) |

Delirium Application (DelApp) score range = 0-10 (10=best possible performance). Note that delirium prevalence is artificially controlled by the case-control design, hence these results have limited clinical value.
