# Supplementary material for: A smartphone-based test for the assessment of attention deficits in delirium: A case-control diagnostic test accuracy study in older hospitalised patients
Source: PLoS One. 2020 Jan 24;15(1):e0227471. doi: 10.1371/journal.pone.0227471 (PMC6980392; doi:10.1371/journal.pone.0227471)
Supplement: S2 Table — (DOCX) [file pone.0227471.s002.docx]

S1 Table. Sensitivity, specificity and Youden’s index for different DelApp score cut-points, for patients with delirium without a formal diagnosis of dementia vs patients without cognitive impairment.

|  | **Delirium (without formal dementia diagnosis) vs. no cognitive impairment** | | |
| --- | --- | --- | --- |
| **DelApp score** | **Sensitivity** | **Specificity** | **Youden’s Index** |
| **1** | 6.9% | 100.0% | 0.07 |
| **2** | 20.7% | 100.0% | 0.21 |
| **3** | 51.7% | 98.5% | 0.50 |
| **4** | 69.0% | 96.9% | 0.66 |
| **5** | 72.4% | 96.9% | 0.69 |
| **6** | 82.8% | 96.9% | 0.80 |
| **7** | **89.7%** | **95.4%** | **0.85** |
| **8** | 96.6% | 84.6% | 0.81 |
| **9** | 100.0% | 1.5% | 0.02 |
| **10** | 100.0% | 0.0% | 0.00 |

Delirium Application (DelApp) score range = 0-10 (10=best possible performance). Patients with a formal diagnosis of dementia were excluded from the delirium group (N=94 removed). Note that the delirium group may still include patients with prior (but undiagnosed) dementia and/or milder cognitive impairments.
