# Supplementary material for: A smartphone-based test for the assessment of attention deficits in delirium: A case-control diagnostic test accuracy study in older hospitalised patients
Source: PLoS One. 2020 Jan 24;15(1):e0227471. doi: 10.1371/journal.pone.0227471 (PMC6980392; doi:10.1371/journal.pone.0227471)
Supplement: S1 Fig — (DOCX) [file pone.0227471.s003.docx]

**S1 Fig. Receiver Operating Characteristic curve for DelApp for detecting DSM-5 delirium in patients without a formal diagnosis of dementia.**


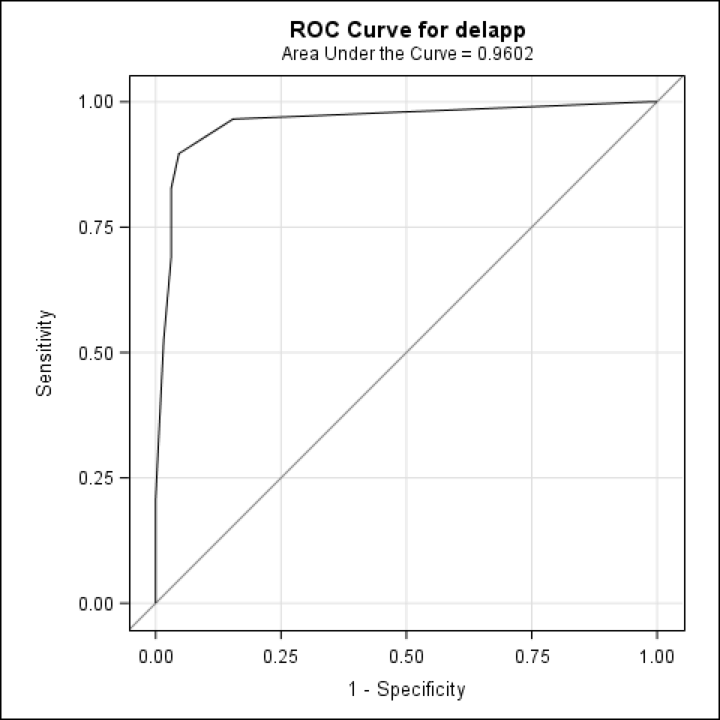


Note: Receiver operating characteristic (ROC) curve analysis was conducted for patients with delirium without a formal diagnosis of dementia (N=29) vs. control patients without cognitive impairment (N=65).
